# Development and application of a clinical core data set for deep brain stimulation in Parkinson’s disease, dystonia or tremor: from data collection to data exchange and data sharing

**DOI:** 10.1186/s42466-024-00362-z

**Published:** 2025-01-30

**Authors:** Anna-Lena Hofmann, Jonas Widmann, Lilly Brandstetter, Udo Selig, Fabian Haug, Julian Haug, Rüdiger Pryss, Jasper Mecklenburg, Andrea Kreichgauer, Philipp Capetian, Christian J. Hartmann, Christian Niklas, Petra Ritter, Patricia Krause, Alfons Schnitzler, Jens Volkmann, Andrea A. Kühn, Peter Heuschmann, Kirsten Haas

**Affiliations:** 1 Institute of Clinical Epidemiology and Biometry, Julius-Maximilians-Universität Würzburg (JMU), Haus D7, Josef-Schneider-Straße 2, 97080 Würzburg, Germany; 2 Institute for Medical Data Science, University Hospital Würzburg, Würzburg, Germany; 3 Department of Neurology and Experimental Neurology, Charité-Universitätsmedizin Berlin, Berlin, Germany; 4 Department of Neurology, University Hospital Würzburg, Würzburg, Germany; 5 Institute of Clinical Neuroscience and Medical Psychology, Medical Faculty and University Hospital Düsseldorf, Heinrich Heine University Düsseldorf, Düsseldorf, Germany; 6 Institute for Medical Informatics, University Hospital Heidelberg, Heidelberg, Germany; 7 Berlin Institute of Health at Charité – Universitätsmedizin Berlin, Berlin, Germany; 8 Department of Neurology With Experimental Neurology, Brain Simulation Section, Charité – Universitätsmedizin Berlin, corporate member of Freie Universität Berlin and Humboldt-Universität Zu Berlin, Berlin, Germany; 9 Bernstein Focus State Dependencies of Learning and Bernstein Center for Computational Neuroscience, Berlin, Germany; 10 Einstein Center for Neuroscience Berlin, Einstein Center Digital Future, Berlin, Germany; 11 Clinical Trial Center, University Hospital Würzburg, Würzburg, Germany

**Keywords:** Clinical core data set, Parkinson’s disease, Dystonia, Tremor, Data exchange

## Abstract

**Background:**

Comprehensive clinical data regarding factors influencing the individual disease course of patients with movement disorders treated with deep brain stimulation might help to better understand disease progression and to develop individualized treatment approaches.

**Methods:**

The clinical core data set was developed by a multidisciplinary working group within the German transregional collaborative research network ReTune. The development followed standardized methodology comprising review of available evidence, a consensus process and performance of the first phase of the study. To ensure high data quality, measures for standardized training, monitoring as well as plausibility and data quality tests were implemented.

**Results:**

The clinical core data set comprises information about medical history, clinical symptoms, information about deep brain stimulation surgery, complications and outcome for the main neurological movement disorders Parkinson’s disease, tremor, and dystonia. Its applicability as well as data exchange and quality control was tested within the first phase of the study in 51 patients from Würzburg.

**Conclusions:**

Within the ReTune project, a standardised clinical core data set for Parkinson’s disease, dystonia and tremor was developed. The collection as well as concepts for the implementation of monitoring and data exchange were elaborated and successfully tested.

*Trial registration number* ClinicalTrials.gov (DRKS-ID: DRKS00031878).

## Background

The clinical trajectories of patients with movement disorders after deep brain stimulation (DBS) vary between individuals and the individual course is difficult to predict [1]. Comprehensive clinical data regarding factors influencing the individual course of the disease as well as identifying trajectories for patients’ outcome might help to better understand disease progression, to develop individualized treatment approaches and to inform translational research projects [2]. For this purpose, clinical data need to be collected in a standardized and comparable way, also allowing data sharing according to the FAIR principles [3] for enabling valid comparative and cross-site data analyses.

The ReTune project is a recently established transregional collaborative research centre (TR-CRC) in Germany focusing on a better understanding of motor network disorders by defining symptom-specific network activity and developing novel concepts for better treatment strategies for network modulation [4].

For ensuring that the clinical data of patients with movement disorders treated with DBS at the various ReTune sites in Berlin, Düsseldorf and Würzburg are collected in a comparable and shareable way, a uniform quality-controlled clinical core data set (CCDS) with common data standards was developed following standardized methodology [5]. Hereby, we describe the development of the CCDS, the technical implementation and quality control measures as well as the results of the first phase of the study.

## Methods

### Development of clinical core data set

The CCDS was defined by a multidisciplinary working group consisting of DBS experts, neurologists, neurosurgeons, and epidemiologist from the participating institutions in ReTune (Berlin, Düsseldorf, Würzburg) by a structured consensus process. The development of the CCDS based on experiences from the QualiPa project, proposing evidence-based quality indicators for DBS in patients with Parkinson’s disease (PD) [5]. In addition, standardized assessment tools used in routine care at the different sites were considered. Based on these information, a proposal for a common CCDS was developed. In a consensus process, the final CCDS was agreed upon by the multidisciplinary working group. The CCDS set is divided into a general part (level 1) and a disease-specific part (level 2). Disease-specific data collection instruments have been defined for PD, dystonia and tremor. Furthermore, the CCDS contains an optional 3-month follow-up and an obligatory 12-month follow-up. In addition, standardized inclusion and exclusion criteria for the different disease entities were defined.

#### Inclusion criteria

The following inclusion criteria for being eligible for inclusion in the CCDS were defined:

- Patients with movement disorders PD, tremor, dystonia (G20-G26: Extrapyramidal diseases and movement disorders) who underwent DBS therapy and who have agreed to participate in the CCDS.
- Patients with PD, tremor, dystonia (G20-G26: Extrapyramidal diseases and movement disorders) eligible for DBS therapy, but who have not opted for DBS therapy and who have agreed to participate in the CCDS (control group).

### Documentation of the CCDS

A detailed concept for documentation and data sharing in a standardized way within ReTune was developed. The CCDS will be documented and stored in a local database at each participating site. As described above, the CCDS comprises assessments and data collected in the clinical routine at the sites. The data collection is technically supported so that there is no double documentation for routine and project data. The local data will then be merged into the central FHIR database of the dotbase platform, which was recently developed by the Charité-Universitätsmedizin for multiaxial data integration for neurological movement disorders and deep brain stimulation [6].

### Technical implementation

The web-based Electronic Data Capture (EDC) system REDCap^©^ is used for collecting the CCDS at two sites (Wuerzburg and Duesseldorf). Before the EDC system was transferred to the productive system, the application went through a testing, training and initiation phase. At Charité, the CCDS is collected directly by integrating the dotbase platform into in the hospital information system (HIS) with direct data transfer into the CCDS. The data is exported via an SSL-encrypted connection between the REDCap^©^ database and the Würzburg-ReTune Web App through predefined API calls of the REDCap^©^ API. Supplementary access security is provided via API tokens and the use of a Github library. The transformation of the CCDS from REDCap^©^ into FHIR format via pre-created matching tables is done by the web app. The core data set variables were assigned to the dotbase variable designations. In addition, a new ID is created in each case. An SSL encrypted connection is established between Würzburg-ReTune Web App and dotbase database to transfer transformed data. There is a supplementary access protection via session cookie stored, which can be obtained by a login call with username and password.

### Use and access regulations

A concept for a standardized Use and Access process for regulating the access to the CCDS was developed and agreed between the participating sites. Researchers request datasets for research purposes via a standardised application for use. The data transfer office at the Institute for Clinical Epidemiology and Biometry (ICE-B) formally reviews the request. The Use & Access Committee consisting of representatives of the project coordination, data transfer office and the data owning site reviews the application. The Use & Access Committee might send required changes to the requesting researcher. In case of approval, a standardised data usage and data deployment agreement is contracted covering the legal and regulatory aspects during and after data transfer. The data transfer office then exports the requested dataset from the central database and performs predetermined and standardised plausibility checks. Afterwards, the dataset is transferred to the requesting researcher in anonymised form.

### Measures for interoperability

For ensuring interoperability also beyond the ReTune project, a cooperation with the Medical Data Model (MDM) portal by the Universities of Münster and Heidelberg was established. The MDM allows standardized sharing of medical data models in eleven different export formats [7]. The content of the electronic Case Report Form (CRF) was published at the MDM (10.21961/mdm:45,864) [8].

### Measures for quality control

Each centre was initiated with dedicated training of study personnel. Additional information material on collecting and documenting the CCDS were provided to the study personnel through training videos, instruction manual and Standard Operating Procedures (SOPs). The topics of recruitment, the reconnaissance process, baseline and follow-up, as well as handling and entering data in the database were explained to the relevant staff. A screening log is used to document eligible study patients based on the inclusion criteria. It is documented how many patients have been screened, are eligible and how many are enrolled in the study. Reasons for non-eligibility or non-participation are recorded. The EDC system contains edit checks and queries during data entry for completeness and plausibility.

Further a concept for remote monitoring was developed. This monitoring concept was defined in consultation between the ReTune sites in Berlin, Düsseldorf and Würzburg. According to this concept a good documentation was defined as 95% of the variables for a patient being filled in completely and correctly. The correctness of a variable was checked using source data verification (SDV). Source data was defined as informed consent, patient record, eCRF, and pseudonymised referral/doctors’ letter. For the monitoring the source data will be provided in a pseudonymised and blackened way using a safe connection fulfilling the data protection requirements. All entries in the CCDS will be compared to the source data by the trained monitor.

According to the concept, the sample size for the SDV is 22 patients. This is based on the assumption that the proportion of incorrect documentation is > 10% and that an incorrect documented patient can be identified during the monitoring with a probability of 90%. To evaluate the practicability of monitoring concept, a test monitoring was performed including two patients. The results of the test monitoring will be presented in this manuscript.

**Fig. 1:**
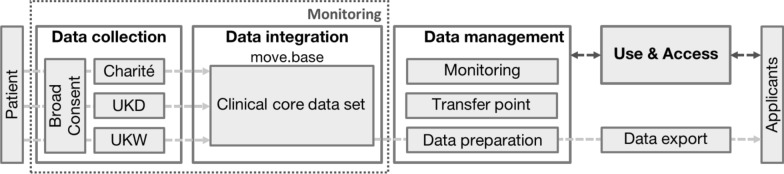
Data flow clinical core data set ReTune

The whole technical implementation of the CCDS is shown in Fig. 1.

## Statistical analyses

The statistical analyses include detailed descriptive and univariate as well as appropriate multivariable analysis methods. Cross-project analyses will be defined by the applicants defined and confirmed by a use and access committee. Patients eligible for DBS treatment are consecutively enrolled at the participating sites. In total, up to 230 patients (Würzburg: 100; Berlin: 30; Düsseldorf: 100) are expected to have an indication for DBS treatment each year at the three sites in Würzburg, Berlin and Düsseldorf. Of these, approximately 147 patients are receiving DBS (Würzburg: 50; Berlin: 27; Düsseldorf 70).

### Ethics and data protection

The study was approved by the ethics committee of the Medical Faculty of the University of Würzburg (EC Würzburg 29/20-me) as well as by the ethics committee in Düsseldorf (Study No.2022–2098) and the Charité-Universitätsmedizin Berlin (CTO 23–0983). Patients or their legal representatives provide written informed consent for participating in the CCDS. A project-specific Broad Consent with a ReTune module was developed based on the template of the Broad Consent V1.6.f of the Medical Informatics Initiative [9]. The data protection concept, the joint controller agreement as well as the regulations for monitoring and use & access were developed and finalized by the participating consortium members. The project is registered in the German Clinical Trials Registry (DRKS00028750).

## Results

### Content of the CCDS

The developed CCDS contains general anamnestic data (Level 1) as well as disease-specific information for patients with PD, dystonia and tremor (Level 2). The content of Level 1 and Level 2 data is shown in Table 1. Of note, the UPDRS I-IV will be tested in the ON and OFF PD medication state. To allow this testing, assessment in the OFF state will be conducted during an inpatient stay. PD patients remained overnight without medication and are assessed in the morning. The content of the CCDS is also available in the MDM portal [8].

**Table 1:** Level 1 general anamnestic patient data

Identification	Pseudonym, Project Affiliation, Center Affiliation
Indication	PD, Dystonia, Tremor, Other Indication
Demographics	Age, Gender, Living Situation, Partnership, Socioeconomic status (highest education)
Observation	Frame of study (study enrolment), Hospital stay baseline (date of admission, date of discharge, main diagnosis, year of first diagnosis, impatient days of stay)
Risk and Lifestyle Factors	Anthropometry (height, weight)
Comorbidities	Previous Clinical diseases (cerebrovascular disease, hypertension, diabetes, chronic pulmonary disease, paralysis, dementia)
Quality of Life	EQ-5D-5L (European Quality of Life 5 Dimensions 5 Level Version) [10], PROMIS-29 (Patient Reported Outcomes Measurement Information System) [11]
Depressions Screening	BDI (II) (Beck-Depression-Inventar Revision II) [12]
Dementia Screening	DRS (Mattis Dementia Rating Scale) [13]
Cognitive Screening	MoCA (Montreal- Cognitive-Assessment) [14]
Balance	FAB (Fullerton Advanced Balance) [15]

Level 2 specific questionnaires for PD, dystonia and tremor (Table 2).

**Table 2:** Level 2 specific questionnaires for Parkinson´s disease, dystonia and tremor)

PD	Dystonia	Tremor
The Unified Parkinson's Disease Rating Scale (UPDRS I-IV) [16]	Burke-Fahn-Marsden Dystonia Rating Scale (BFMDRS) [17]	Scale for the Assessment and Rating of Ataxia (SARA) [18]
Hoehn and Yahr Scale [19]	Toronto Western Spasmodic Torticollis Rating Scale (TWSTRS) [20, 21]	Fahn-Tolosa Marin Tremor Rating Scale (FTMTRS) [22]
The Parkinson's Disease Questionnaire (PDQ-39) [23]		
Questionnaire for Impulsive-Compulsive Disorders (QUIP) [24]		
Ardouin Scale of Behaviour [25]		

### Technical implementation

The data transfer from Würzburg to Berlin was successfully tested with 44 patients. A tailored web app (ASP.NET Core) was implemented to transfer data from the REDCap system to the dotbase platform. This web app is deployed on a server of the participating site. This and other security components (SSL, API token, login, session cookie: SSL standard HTTPS connection. API token provided by RedCap. Session cookie provided by dotbase environment.) ensure that the data is not sent or processed unencrypted (asymmetric, public kex) outside the hospital network. The transmission process (ASP.NET Core, implemented in C#) consisted of 3 basic components: 1. exporting the data from the REDCap system; 2. formatting the data from the REDCap format into the FHIR format using predefined matching tables; 3. transfer of the formatted data into the dotbase platform.

### Data collection

At the Würzburg site, 51 patients were enrolled in the ReTune study between February and September 2023. The response rate of eligible patients was 71%. These patients were documented according to the local CCDS. Level 1 and Level 2 of the CCDS could be surveyed almost completely. Data collection and entry into CRF takes about 3.5 h per patient. As there were no follow-up visits during this time period, the data collection was limited to the baseline survey. Due to organizational circumstances, the details on surgical procedures are only available with a delay, therefore details are not available yet. Exemplary, the characteristics of these patients are described in Table 3.

**Table 3:** Patients characteristics (Level1) and results of specific Questionnaires (Level 2)

Population characteristics (Level 1)		Specific questionnaires (Level 2)	
*Population Characteristics (Level 1) N* = *51*			
Age mean (SD)	64.3 (9.0)	Parkinson´s disease N = 42	
*Gender n (%)*			
Female	30 (59)	*MDS-UPDRS*	
Male	14 (27)	Part I Summary score mean (SD)	3.2 (3.4)
Missing	7 (14)	Part II Summary score (med on) mean (SD)	16.6 (9.5)
*Education n (%)*		Part II Summary score (med off) mean (SD)	17.8 (8.9)
High	13 (25)	Part III Summary score (med on) mean (SD)	17.4 (9.6)
Med	17 (33)	Part III Summary score (med off) mean (SD)	39.2 (16.3)
Low	10 (20)	Part IV Summary score mean (SD)	6.8 (5.0)
Other	1 (2.0)		
Missing	10 (20)	*Hoehn and Yahr scale*	
*Disorder n (%)*		Hoehn and Yahr summary score mean (SD)	2.2 (0.9)
Parkinson	42 (82)		
Dystonia	5 (9.8)	*The Parkinson's Disease Questionnaire (PDQ-39)*	
Tremor	4 (7.8)	PDQ-39 summary score mean (SD)	23.9 (13.7)
*Living Situation n (%)*			
Independent at home	37 (73)	*The questionnaire for impulsive-compulsive disorders (QUIP)*	
Home nursing care	2 (3.9)	Subscore gambling mean (SD)	1.1 (2.8)
Nursing home	1 (2.0)	Subscore sex mean (SD)	1.8 (2.3)
Missing	11 (22)	Subscore buying mean (SD)	1.4 (2.5)
*Partnership n (%)*		Subscore eating mean (SD)	2.6 (3.2)
Yes	36 (71)	Subscore punding mean (SD)	2.4 (2.7)
No	5 (9.8)	Subscore hobbyism mean (SD)	1.7 (2.7)
Missing	10 (20)	Subscore medication mean (SD)	1.9 (2.7)
*EQ-5D-5L*		Dystonia N = 5	
EQ-5D-5L Index mean (SD)	0.73 (0.16)		
		*Burke-Fahn-Marsden Dystonia Rating Scale (BFMDRS)*	
*Beck's Depression Inventory*		Movement scale mean (SD)	11.0 (6.2)
Summary score mean (SD)	10.6 (6.4)	Dystonia disability scale mean (SD)	4.0 (4.5)
No depression n (%)	9 (17.6)		
Light depression n (%)	24 (47.1)	*Toronto Western Spasmodic Torticollis Rating Scale (TWSTRS)*	
Moderate depression n (%)	13 (25.5)	Torticollis severity scale mean (SD)	15.5 (9.3)
Severe depression n (%)	4 (7.8)	Disability scale mean (SD)	13.6 (3.3)
Missing n (%)	1 (2.0)	Pain scale mean (SD)	3.5 (2.1)
		Total Score mean (SD)	8.9 (5.3)
*Montreal-Cognitive-Assessement (MoCa)*			
MoCa summary score mean (SD)	27.1 (3.0)	Tremor N = 4	
*The Mattis Dementia Rating Scale (MDRS)*		*Scale for the Assessment and Rating of Ataxia (SARA)*	
MDRS summary score mean (SD)	140.0 (5.1)	Gait score mean (SD)	1.5 (1.3)
		Stance score mean (SD)	1.0 (1.2)
		Sitting score mean (SD)	0.0 (0.0)
		Speech disturbance score mean (SD)	1.8 (1.3)
		Finger chase score mean (SD)	0.9 (1.0)
		Nose-finger test score mean (SD)	2.5 (0.7)
		Fast alternating hand movements mean (SD)	1.3 (1.2)
		Heel-shin slide score mean (SD)	0.9 (1.0)
		*Fahn-Tolosa Marin Tremor Rating Scale (FTMTRS)*	
		Part A score mean (SD)	20.3 (14.4)
		Part B score mean (SD)	22.5 (10.2)
		Part C score mean (SD)	12.5 (6.8)
		Total score mean (SD)	55.3 (28.9)

### Quality measures

A first remote monitoring was performed at the collecting site in Würzburg in two exemplarily patients (one with dystonia and one with PD) with complete documentation. The entries in REDCap were analysed by SDV using clinical records. Furthermore, the completeness and the quality of the entries in REDCap were evaluated. The results of the first monitoring in Würzburg show a high completion rate with 92% of items completed.

## Discussion

In the course of the ReTune project, a CCDS set with general patient information as well as PD, dystonia and tremor specific elements was successfully developed allowing the standardized collection and exchange of data at different sites with high expertise in DBS treatment within the setting of the ReTune project (Berlin, Düsseldorf and Würzburg).

A central database structure for storing the data was implemented and quality measures were established comprising training, remote monitoring and quality control of the data. The data exchange and the concept for automated export from the local centres to the central dotbase database was achieved by the interoperable FHIR standard. The interoperability for the use of the CCDS for other research projects is ensured by the metadata registry MDM. In addition, measures for ensuring data access and data sharing according to the FAIR principles were proposed including also dedicated Use & Access procedures. The developed concepts were approved by all participating sites. The application of the developed tools was successful tested within a first phase of the study at one site.

World-wide, several PD registries exist (e.g. [26–35]). However, these registries did not focus specifically on DBS patients. Furthermore, several longitudinal studies and systematic reviews on DBS outcomes were conducted [36–42]. The observed PD populations were comparable regarding the mean age at DBS (ranging from 53–63 years) and mean Hoehn and Yahr stage (ranging from 2.0–3.1) [36–41]. Additionally, mean MDS-UPDRS scores were within the range of other studies (UPDRS I range 2.8–3.3, UPDRS II (med off) 17.5–19.6, UPDRS III (med off) 29.2–75.0, UPDRS III (med on) 12.5–24.8, UPDRS IV 5.1–8.8). The PDQ-39 was rarely used in studies of other registries and the reported scores found to be higher (29.6 and 31.3) [36–41]. Huge difference for the ratio of male and female DBS patients was observed (between 30 and 93% males) compared to our study population lining up with the lower percentages of male DBS patients [36–41]. Furthermore, a review on DBS outcomes in dystonia patients reported higher TWSTRS scores than in dystonia patients in our study population (severity score 22.0, disability scale 19.3, pain scale 11.8, total score 50.8) [42].

The jointly developed infrastructure is feasible to perform central remote monitoring and to exchange the standardized data. So the quality of data collected in clinical routine at different sites will be improved. Researchers would be able to obtain a well-defined data set to answer further research question via the Use & Access process. This promotes collaborative research and facilitates further usage of data. One challenge is to connect the different EDC systems at the different sites. Common data standards and interoperability have been achieved by using the FHIR format and modelling via the MDM portal [8].

The monitoring performed locally at the Würzburg site has shown a high completion rate and data quality (92%) as surgery details and the corresponding outcomes could already be documented. For patients with pending surgery for implantation of the electrodes, these details could not yet be documented, nor could the outcome parameters based on them. Overall, the monitoring proved to be very helpful for the study nurse to discuss outstanding questions. This enables a further increase in data quality and completeness. The analysis of the pilot phase of the study has shown a high willingness to participate on the part of the patients, a high documentation rate and data quality as well as possibilities for joint data sharing and exchange due to multidisciplinary cooperation, a comprehensive training concept and interoperability standards.

In the future, additional sites could be included in the core data set collection. In addition, expansion to include electrophysiological and imaging data is possible [19, 43]. It is planned to expand this dataset and introduce a unified platform that directly integrates multiaxial data for all clinical sites. The core clinical dataset will be expanded to include DBS stimulation parameters, biomaterial, imaging parameters, electrophysiological data, and milestones of disease progression such as falls and movement patterns, as well as digital phenotyping and ecological momentary assessment. The platform will be connected to mobile applications to collect data directly from the patients. It is planned to extend the dotbase platform to Düsseldorf and Würzburg IT infrastructure by integrating the platform directly within hospital information systems at these sites. The established register can additionally be used as trial-ready cohort [to identify patients eligible] for initial proof-of-concept studies or future clinical trials. Furthermore, the ReTune project might allow the sustention of the established register within routine care.

### Strengths and limitations

A high willingness of patients to participate (71%) was observed. On the basis of using a comprehensive training concept, the site study personnel were well prepared for their tasks during the study implementation. By means of training videos, a training manual and SOPs, the topics of recruitment, the reconnaissance process, baseline and follow-up, as well as handling and entering data in the database were trained. A test monitoring in Würzburg shows the good feasibility of data entry and quality.

A conspicuous advantage inherent in the implemented data transfer is its prompt adaptability within the web application to accommodate alterations in the CCDS. This agility is facilitated by the application's generic structure, enabling facile modifications or expansions through the mere adaptation of the matching tables.

However, there are still limitations, as so far there is only information on the availability of imaging and electrophysiological data, but no linking to the raw or processed data. Future projects should therefore focus on establishing this linkage and on compliant storage of those more complex data types that cannot be stored as simple text elements and hence come with additional requirements.

## Conclusions

Within the ReTune project, we successfully established infrastructures for enabling a standardized collection of clinical data in PD patients as well as in patients with essential tremor and dystonia treated with DBS resulting in the provision of a comprehensive, well-defined clinical data set. Moreover, measures (workflows) that allow access to patient data in a standardized way were developed. Those elements will facilitate and broaden the usage of data as they could be rolled out to build a national disease specific register and they could be used as a blueprint for collecting data in other movement disorders by adapting modules specific for those diseases. Furthermore the dataset of the established cohort might also build the framework for developing complex interventional studies to further improve patients´ health care.

## Data Availability

In keeping with prevailing current practice, Movement Disorders expects that data supporting the results in the paper will be archived in an appropriate public repository. Authors are required to provide a data availability statement to describe the availability or the absence of shared data. When data have been shared, authors are required to include in their data availability statement a link to the repository they have used, and to cite the data they have shared. Whenever possible the scripts and other artefacts used to generate the analyses presented in the paper should also be publicly archived. Exceptions are made if sharing data compromises ethical standards or legal requirements. Please refer to https://authorservices.wiley.com/author-resources/Journal-Authors/open-access/data-sharing-citation/data-sharing-policy.html. The data of this study are available upon request from the Use & Access Committee of the SFB TRR ReTune 295.

## Abbreviations

CCDS: Clinical core data set
CRF: Electronic case report form
DBS: Ceep brain stimulation
EDC: Electronic data capture
HIS: Hospital information system
ICE-B: Institute for Clinical Epidemiology and Biometry
MDM: Medical data model
PD: Parkinson’s disease
SDV: Source data verification
SOPs: Standard operating procedures
TR-CRC: Transregional collaborative research centre
